# Recommendations for radiographers and radiation therapists drawn from an analysis of errors on Australian Radiation Incident Registers

**DOI:** 10.1002/jmrs.206

**Published:** 2017-01-05

**Authors:** Gary Denham, Nicole Page

**Affiliations:** ^1^ Hunter New England Health Taree New South Wales Australia; ^2^ Calvary Mater Newcastle New South Wales Australia

**Keywords:** Mandatory reporting, patient safety, radiation protection, risk management, safety

## Abstract

**Introduction:**

The Australian Radiation Protection and Nuclear Safety Agency (ARPANSA) collect reported incidents for inclusion in the Australian Radiation Incident Register (ARIR), a database of radiation incident reports that occur within Australia. While the information on previous radiation incidents is available, there is little information on the lessons that can be learned from those past incidents to help prevent the same errors reoccurring. The aims of the study were to investigate what radiation incident registers are publicly available in Australia and to utilise the information contained within the ARIR and any other state or territory radiation protection authority registers to make recommendations for radiographers and radiation therapists to prevent future adverse events.

**Methods:**

A search was conducted to locate what radiation incident registers within Australia were available to the public. All adverse events from 2003 to 2014 were compiled into a spreadsheet for analysis. An error‐type classification taxonomy was used to classify the adverse events. Conclusions were drawn from the determined causes to make recommendations to change work practices in an attempt to prevent similar adverse events reoccurring.

**Results:**

Incident registers were located from New South Wales, South Australia, Tasmania, Victoria and Western Australia. Radiography represented 76% (243) of the adverse events. A vast majority of the incidents were a failure to comply with time‐out protocols (77%, 248).

**Conclusion:**

There are several radiation adverse event registers publicly available to utilise in Australia. All departments need to adopt and strictly adhere to time‐out protocols. This in conjunction with the other recommendations in this article has the potential to dramatically reduce radiation adverse events.

## Introduction

When adverse events occur in the medical radiation science (MRS) profession, the incident is investigated and the incident along with the findings of the investigation is reported to the relevant state or territory government radiation protection authority. The Australian Radiation Protection and Nuclear Safety Agency (ARPANSA) collect reported incidents for inclusion in the Australian Radiation Incident Register (ARIR), a database of radiation incident reports that occur within Australia.^1^

The Australian Radiation Protection and Nuclear Safety Agency compile radiation incident reports to produce annual summary reports of the ARIR. The World Alliance for Patient Safety states: ‘The primary purpose of patient safety reporting systems is to learn from experience. It is important to note that reporting in itself does not improve safety. It is the response to reports that leads to change’.^2^ ^(p12)^ For the ARIR to be an effective tool to reduce radiation incidents, the information from past experiences contained within the annual summary reports must be utilised as a learning tool to change systems and behaviours.

Schedule 13 of the National Directory of Radiation Protection specifies the incidents that must be reported to ARPANSA for inclusion in the ARIR. Radiographers and radiation therapists have to report incidents in the following circumstances:

- Any diagnostic procedure other than as prescribed by the medical practitioner.
- Any diagnostic procedure resulting in an observable acute radiation effect.
- Any therapeutic treatment delivered to either the wrong patient or the wrong tissue or using the wrong radiopharmaceutical.
- When during administration of a therapeutic dose of radiation from a radiation apparatus or a sealed radioactive source, the dose delivered differs from the total prescribed treatment dose by more than 10%.^3^ ^(p37)^

One of the objectives of the ARIR when it was established was ‘through appropriate publications, to provide feedback and guidance to users of radiation on preventing or limiting the consequences of radiation accidents’.^4^ ^(¶4)^ The annual summary reports are the only documents to be released by ARPANSA from the information contained within the ARIR. Summary reports from 2012 onwards contain additional information on the causes of the incidents but are not detailed enough to draw recommendations for improvements in safety. ARPANSA plan to include more detailed information in future annual summary reports on post‐incident follow‐up and lessons learnt.^4^ While the information on previous radiation incidents is available, there is little information on the lessons that can be learned from those past incidents to help prevent the same errors occurring in future.

The aims of the study were:

- To investigate if any radiation protection authority incident registers within Australia are available to the public.
- Utilise the information contained within the ARIR and any state or territory radiation protection authority registers to make recommendations for radiographers and radiation therapists to prevent future adverse events.

## Methods

As this study involved publicly available anonymised data, ethics approval was not required. A search was conducted from January 2015 until March 2015 of all state and territory health department's, environment authority's and radiation authority's websites (*keywords*: ‘Radiation’, ‘Radiation incidents’, ‘Radiation accident’) to locate what radiation incident registers within Australia were available to the public. Authorities responsible for radiation in each state and territory were contacted via e‐mail to confirm if radiation incidents data were publicly available.

All diagnostic radiography (DR) and radiation therapy (RT) adverse events available from the state and territory registers along with those in the ARIR that occurred between 2003 and 2014 were compiled into a spreadsheet for analysis. Incidents were excluded if the researchers concluded there was inadequate information to determine a cause or were deemed to be no fault of the MRS personnel involved (Table 1).

**Table 1:** Examples of incidents excluded from analysis

Reasons for exclusion	Example
Inadequate information to determine cause	‘The patient received two unscheduled CT scans, resulting in an estimated total effective dose to the patient of 25 mSv’^5^ ^(p1)^
No fault of MRS personnel	‘The patient was not aware of being pregnant or replied that she was not pregnant when asked at the time’^6^ ^(p1)^

MRS, medical radiation science; CT, computerised tomography.

An error‐ type classification taxonomy^2^ was devised by the researchers to classify the adverse events (Table 2). In the 2012 and 2013 annual summary reports the vast majority of incidents are classified as human error, 70% and 69% respectively.^7^, ^8^ Technically, one could argue all radiation adverse events are due to human error, but a greater number of categories were required to perform a more in‐depth analysis to draw conclusions from. To achieve this, the incidents were categorised into one of the 17 devised classifications. The researchers analysed and classified all adverse events independently. Any incidents that were not classified unanimously were decided by discussion and consensus.

**Table 2:** The classification of adverse events by determined cause. Each adverse event was allocated into 1 of 17 sub‐categories (shaded in blue)

Classification	Definition
Booking Procedures	Errors that occur due to the systems in place to request (or cancel) a procedure.
Internal systems	These errors occur before reaching the MRS professional. They occur either externally to the department (e.g. electronic x‐ray requests) or within (e.g. department reception) and would not likely be detected during a correctly performed time‐out procedure.
Non‐original request form	Errors that occur due to the use of any type of duplicate or non‐original referral/prescription form.
Failure to comply with time‐out protocol	Any error that occurs that would reasonably be expected to be detected and thwarted by carrying out a correctly performed ‘correct patient, correct site and correct procedure’ time‐out protocol and pregnancy/breastfeeding check.
Failure to comply with time‐out protocol. Patient	Any error that involves a procedure performed on the incorrect patient (or foetus) that would reasonably be detected by a time‐out protocol.
Closed questions	Due to closed questions being asked when identifying a patient.
Non‐compliance	Due to the time‐out protocol not being performed.
Request error	Due to incorrect patient details on the request/prescription form that would be reasonably detected by a time‐out protocol (e.g. an incorrect patient sticker).
Pregnancy/breastfeeding	Due to a pregnancy/breastfeeding check not being performed.
Failure to comply with time‐out protocol. Procedure	Any error that involves the incorrect procedure being performed that would reasonably be detected by a time‐out protocol.
Handover	When an incorrect procedure is performed due to handover from one staff member to another where incorrect and or inadequate information is passed on or the new staff member fails to make the appropriate checks before proceeding with the procedure.
Human error	Any error that occurs when the incorrect procedure is performed and no other category applies (e.g. radiographer is distracted and forgets to connect pressure injector to patient's cannula).
Internal systems	Due to procedures or systems within the practice that contributed to the incorrect procedure being performed.
Non‐compliance	Due to the procedure not being checked on the request/prescription or matched to patient presentation.
Request error	Due to an error on the request form.
Request form ambiguity	When the incorrect procedure is performed due to ambiguity of the request/prescription.
Side	Any error that involves the correct procedure performed on the correct patient but performed on the opposing side.
Other	All other categories.
Inadequate student/intern supervision	Errors performed by students or interns under the supervision of qualified MRS personnel.
Inadequate training	Due to unfamiliarity of software, equipment or procedures.
Unintentional radiation exposure to staff or public	The unintentional irradiation of staff or members of the public.
Unlicensed use of radiation apparatus	The irradiation of any individual due to unlicensed operation of a radiation source.

MRS, medical radiation science.

Each researcher then analysed the adverse events from their own MRS specialty to find reoccurring causes within each classification. Conclusions were drawn from the determined causes to make recommendations to change work practices in an attempt to prevent similar adverse events occurring in the future.

## Results

Apart from the ARIR, five publicly available radiation incident registers were located from New South Wales (NSW), South Australia (SA), Tasmania (TAS), Victoria (VIC) and Western Australia (WA). These incident registers are contained within annual reports of the relevant state government authority (Table 3). Incident registers for the Northern Territory (NT) and Queensland (QLD) were not located, and replies to e‐mails were not received from these two jurisdictions, however the number of radiation incidents in NT are listed in the NT Department of Health Annual Reports.^9^

**Table 3:** Publicly available radiation incident registers within Australia

Authority	Document	Web address
Australian Radiation Protection and Nuclear Safety Agency	Australian Radiation Incident Register Annual Summary Reports	http://arpansa.gov.au/radiationprotection/arir/arir-reports.cfm
New South Wales Environment Protection Authority	Radiation Advisory Council Annual Reports	https://www.epa.nsw.gov.au/radiation/radiationpubs.htm
South Australian Environment Protection Authority	Annual Reports on the administration of the Radiation Protection and Control Act 1982 (within the EPA Annual Reports)	http://www.epa.sa.gov.au/data_and_publications/search-documents?q=%27epa+annual+report%27&published=&category=&doctype=
Tasmania Department of Health & Human Services	Annual Reports on the Operation of the Radiation Protection Act 2005	http://www.dhhs.tas.gov.au/publichealth/radiation/publications2/reports
Victoria Department of Health	Radiation Act 2005 Annual Reports	https://www2.health.vic.gov.au/about/publications/annualreports
Western Australia Radiological Council	Radiological Council of Western Australia Annual Reports	http://www.radiologicalcouncil.wa.gov.au/

A total of 356 adverse events were derived from the incident registers between 2003 and 2013 that the researchers determined a cause could be concluded (262 DR, 94 RT). There were 35 incidents (19 DR, 16 RT) determined to be duplicated in both a state authority register and the ARIR, so these were considered to count as one incident for the purposes of this study. This left 321 incidents (243 DR, 78 RT) being subject to further scrutiny.

DR represented 76% of the 321 adverse events. A vast majority of the incidents were a failure to comply with time‐out protocols (77%) and half of those were due to non‐compliance where the time‐out protocol was not carried out. The categorisation of the total number of incidents included in the study is detailed in Table 4.

**Table 4:** Breakdown of incidents into the determined cause categories

Category	DR incidents *n* (%)	RT incidents *n* (%)	Total DR and RT incidents *n* (%)
Booking procedures	48 (19.75%)	2 (2.56%)	50 (15.58%)
Internal systems	29 (11.93%)	2 (2.56%)	31 (9.66%)
Non‐original request form	19 (7.82%)	–	19 (5.92%)
Failure to comply with time‐out protocol	177 (72.84%)	71 (91.03%)	248 (77.26%)
Failure to comply with time‐out protocol: Patient	69 (28.40%)	5 (6.41%)	74 (23.05%)
Closed questions	7 (2.88%)	–	7 (2.18%)
Non‐compliance	53 (21.81%)	5 (6.41%)	58 (18.07%)
Request error	5 (2.06%)	–	5 (1.56%)
Pregnancy/breastfeeding	4 (1.65%)	–	4 (1.25%)
Failure to comply with time‐out protocol: Procedure	108 (44.44%)	66 (84.62%)	174 (54.21%)
Handover	7 (2.88%)	–	7 (2.18%)
Human error	34 (13.99%)	38 (48.72%)	72 (22.43%)
Internal systems	2 (0.82%)	6 (7.69%)	8 (2.49%)
Non‐compliance	52 (21.40%)	14 (17.95%)	66 (20.56%)
Request error	–	3 (3.85%)	3 (0.93%)
Request form ambiguity	8 (3.29%)	2 (2.56%)	10 (3.12%)
Side	5 (2.06%)	3 (3.85%)	8 (2.49%)
Other	18 (7.41%)	5 (6.41%)	23 (7.17%)
Inadequate student/intern supervision	5 (2.06%)	1 (1.28%)	6 (1.87%)
Inadequate training	7 (2.88%)	1 (1.28%)	8 (2.49%)
Unintentional radiation exposure to staff or public	3 (1.23%)	3 (3.85%)	6 (1.87%)
Unlicensed use of radiation apparatus	3 (1.23%)	–	3 (0.93%)
Total	243	78	321

DR, diagnostic radiology; RT, radiation therapy.

## Discussion

It is not surprising that DR errors outnumber those of RT by approximately 3:1. Radiation therapists often work in pairs checking each other's work and the procedure times are longer resulting in fewer procedures performed. RT also has more quality assurance checks due to the higher risks associated with greater radiation doses.

The use of the ‘correct patient, correct site and correct procedure’ protocol for operating theatres was mandated by Australian health ministers in 2004.^10^ Similar protocols were developed for DR and RT in 2008 but were never mandated.^11^ Patient identification is, however, one of the standards (standard 5) in The National Safety and Quality Health Service Standards that ‘are a critical component of the Australian Health Services Safety and Quality Accreditation Scheme’^10^ ^(p2)^ and the correct patient, correct site and correct procedure protocol is a way of meeting the mandatory requirements of that standard.^12^ MRS professionals also have an obligation under the Professional Practice Standards of the Australian Society of Medical Imaging and Radiation Therapy (previously the Australian Institute of Radiography) to follow these time‐out procedures.^13^ Despite errors associated with patient identification being difficult to eliminate, a system of patient and procedure matching like the one proposed by Danaher et al.^14^ demonstrated significant improvements can be achieved.

Underreporting of incidents has been well documented in medicine^15^, ^16^, ^17^ and is an ongoing issue in MRS as well.^14^, ^18^, ^19^, ^20^ The various reporting requirements of the state jurisdictions within Australia also contribute to the underreporting of adverse events.^18^ James Reason, the esteemed author in the field of human error, argues that for error reporting to make a meaningful contribution to safety the culture of blame and culpability must be replaced with a reporting culture.^21^ The fear of blame and reprisals when reporting adverse events is well documented in medicine^15^, ^17^, ^22^ and may be a reason for underreporting in MRS. This is an area for possible further research.

This study identified several other publicly available radiation registers apart from the ARIR. Causes of incidents are easier to determine the more information that is made available; the NSW register generally contained more information than the ARIR, while the WA and VIC registers contained far more detailed information on each incident that makes those registers an invaluable learning resource. The Radiology Events Register (RaER) is a voluntary radiology incidents database that contains case studies that are also useful learning resources.^23^ These case studies along with the state registers and the ARIR should be utilised by all departments in their continuing professional development programme to learn from the errors that have occurred at other institutions.

Several trends were identified in the analysis of the incidents to draw recommendations from. Almost 39% of the adverse events were due to non‐compliance in which the description of the incident clearly stated the time‐out protocol was not performed. All facilities must develop, practice, perfect and monitor the use of time‐out protocols appropriate to the organisation. The implementation of this one recommendation addresses numerous error classifications in this study, such as the use of closed questions, and has the potential to drastically reduce the rate of adverse events that occur in DR and RT.

Booking procedures also had several reoccurring causes identified. There were numerous incidents in DR of two requests being submitted for the same examination and being completed twice. Incidents in both DR and RT of examinations being cancelled and performed anyway. Examinations in DR were also rescheduled and performed on both occasions. All practices need to examine their clerical procedures to look for deficiencies of this kind. Many patient scheduling software packages incorporate warnings for duplicated examinations within preceding time frames to assist with this. Another reoccurring cause of incidents in DR was the use of faxed request forms and the examination being performed again with the original form. Strict policies and procedures for the use of faxed request forms need to be implemented to reduce these types of errors occurring.

There were several RT incidents that involved the use of proposed instead of actual prescriptions or fields for treatment. These errors can be prevented by having clearly defined protocols where the radiation oncologist signs off all information/scans to be used for the patient treatment at predefined stages throughout the simulation and planning process, and any preliminary data not utilised should be deleted or filed in non‐clinical folders so it cannot be confused with the actual treatment plan.

A number of incidents occurred in DR where the incorrect patient identification label was affixed to the request form. A considerable portion of identification errors are due to the use of stickers and this is unlikely to be eliminated with the use of electronic requesting,^14^ so this type of error highlights the importance of checking the procedure as part of the time‐out protocol.

The sub‐category of Failure to Comply with Time‐Out Protocols: Procedure: Human Error had numerous causes but some reoccurring causes were identified. The reoccurring causes along with the recommendations to prevent them are detailed in Table 5.

**Table 5:** Reoccurring causes in the ‘human error’ category and recommendations for prevention

Modality	Cause	Recommendations
RT	17 incidents involve a geographical miss of isocentre placement	Verification of treatment position with daily imaging, tolerances and overrides by both radiation therapists. Establish ‘no interruption zones’ around the treatment console. Documentation of patient position and labelling of stabilisation devices signed off by both radiation therapists.
RT	9 incidents involve the incorrect manual transcript/entry	Create checklist for transcripts, computer entries and calculations pre‐treatment.
DR	6 incidents involve the wrong CT scan protocol being selected	Have clearly defined protocols with as little abbreviation as possible with similar protocols separated.
DR	4 incidents involve the images being deleted or not sent to PACS	Include automatic saving of raw CT image data or fine slice images to PACS in every protocol.
RT	3 incidents involve the incorrect interpretation of the pre‐image	Establish guidelines to ensure enough surrounding anatomy is visible in order to accurately determine the isocentre placement.
DR	3 incidents where the paperwork was mixed up with another	Have systems in place that have one patient's paperwork in the work space at one time and allocated spaces for stages ‘to do’, ‘done’, etc.

DR, diagnostic radiology; RT, radiation therapy; CT, computerised tomography; PACS, picture archiving and communication system.

Although the aims of the research have been achieved, there are limitations to this study. The authors acknowledge they are not experts in the field of occupational or nuclear safety and the recommendations are those from people working in and understanding the processes of the profession. Many more incidents from the ARIR could have been analysed if anonymised data were sought from ARPANSA instead of the brief descriptions in the annual summary reports; however, the authors set out to use publicly available data throughout Australia to determine if MRS professionals could utilise the data to make useful suggestions to prevent future adverse events.

## Conclusion

As MRS professionals, we have an obligation to minimise and prevent adverse events when interacting with our patients. One way to achieve this is to learn from previous errors that have occurred and there are several radiation adverse event registers publicly available in Australia that can be utilised for that purpose. The knowledge obtained from these registers can help prevent future adverse events occurring.

An overwhelming percentage of adverse events in radiography and radiation therapy occur due to the failure to comply with time‐out protocols. All departments need to adopt and strictly adhere to the protocols mandated by organisations Australia wide. This in conjunction with the other recommendations in this article that has the potential to dramatically reduce radiation adverse events and improve safety for staff, patients and members of the public alike.

## Conflict of Interest

The authors declare no conflict of interest.
